# Sequential production of motor-action verb subtypes in Parkinson's disease patients

**DOI:** 10.1590/1980-5764-DN-2022-0027

**Published:** 2023-02-06

**Authors:** Mireya Chávez-Oliveros, Julio César Flores-Lázaro, Haydee Durán Meza, Wendy Ramírez-Burgos

**Affiliations:** 1 Instituto Nacional de Neurología y Neurocirugía, Departamento de Neuropsicología, Ciudad de México, Mexico.; 2 Secretaria de Salud de Mexico, Hospital Psiquiátrico Infantil Dr. Juan N. Navarro, Servicios de Atención Psiquiátrica, Ciudad de México, Mexico.; 3 Universidad Nacional Autónoma de México, Facultad de Psicología, Ciudad de México, Mexico.; 4 Universidad Nacional Autónoma de México, Facultad de Estudios Superiores Zaragoza, Ciudad de México, México.

**Keywords:** Parkinson's Disease, Aging, Executive Functions, Language, Doença de Parkinson, Envelhecimento, Função Executiva, Idioma

## Abstract

Motor-action verbs (MAVs) production and comprehension are compromised in patients with Parkinson's disease (PD).

**Objectives:**

The aim of this study was to characterize the sequential production of three subtypes of MAVs in PD patients: whole body (e.g., *run*), specific body part (e.g., *kick*), and instrumental (e.g., *saw*). This study also aimed to identify the production characteristics for each of the two main phases in fluency performance: selection (initial abundant item production) and retrieval (more paced and scarce production).

**Methods:**

This study involved a group of 20 nondemented, on-medication PD patients, with an average age of 66.59 years (standard deviation = 4.13), and a comparison group (CG) of 20 normal elderly individuals, matched by years of education and controlled for cognitive performance and depression. Both groups performed a classical verb fluency task. Sequential word-by-word analyses were conducted.

**Results:**

Significant differences were found at the initial production of whole-body MAVs and the overall production of instrumental verbs (both measures were lower in the PD group). A repeated-measures analysis of variance confirmed the linear CG performance and the quadratic PD performance.

**Conclusions:**

PD patients present altered production of whole-body and instrumental MAVs. This proposal for the semantic sequential analysis of motor verbs deserves further investigation, as a new methodology for the evaluation of fluency performance in motor-related disease.

## RESUMO

A produção e a compreensão das ações motoras (MAVs) estão comprometidas em pacientes com doença de Parkinson (DP).

**Objetivos:**

Caracterizar a produção sequencial de três subtipos de verbos de MAVs: corpo inteiro (por exemplo, corre), parte corporal específica (por exemplo, chute) e instrumental (por exemplo, serra) em pacientes com DP. Identificar as características de produção para cada uma das duas principais fases em desempenho de fluência: seleção (produção inicial abundante de itens) e recuperação (produção mais acelerada e escassa).

**Métodos:**

Um grupo de 20 pacientes com DP não demência, com idade média de 66,59 (desvio padrão — DP= 4,13), e um grupo de comparação (GC) de 20 idosos normais, dísticos com anos de estudo e controle para desempenho cognitivo e depressão. Ambos os grupos realizaram uma tarefa clássica de fluência de verbo. Foram realizadas análises sequenciais palavra por palavra.

**Resultados:**

Diferenças significativas foram encontradas na produção inicial de MAVs de corpo inteiro e na produção global de verbos instrumentais (ambas as medidas foram menores no grupo PD). Uma medida repetida na análise de variância (ANOVA) confirmou o desempenho linear de CG e o desempenho quadrático de DP.

**Conclusões:**

Os pacientes com DP apresentam produção alterada de MAVs corporais inteiras e instrumentais. Esta proposta para a análise sequencial semântica dos verbos motores merece uma nova investigação, como uma nova metodologia para a avaliação do desempenho da fluência em doenças motoras.

## INTRODUCTION

Action fluency (verb production) and action language processing (comprehension, semantic comparison, etc.) are compromised in patients with Parkinson's disease (PD)^1^. Actions performed by specific parts of the body are particularly affected, which can be mainly explained by dopamine deficiency: Herrera et al.^2^ studied the on/off medication effect (levodopa) on verbal fluency performance in a group of 34 nondemented PD patients and concluded that patients on medication produced a greater number of verbs with high motor specificity (e.g., *sew, knit,* and *bounce*) than those with low motor specificity (e.g., *swim, run,* and *sleep*).

### Subtypes of motor-action verbs

In the literature, three types of motor-action verbs (MAVs) have been described: whole body (WBAVs), specific body verbs (SBAVs), and instrumental verbs (InstVs), where each type of verb has a different neuropsychological and functional neuroimaging correlate^3^:

Whole-body action verbs: actions performed by or with most of the body, such as running, swimming, and jumping^4^

Specific body part action verbs: actions performed by specific body parts, such as kicking, biting, and blinking^5^

Instrumental verbs: actions performed using an instrument or an object, such as cutting and sawing^6^

### Neuropsychology of verbal fluency paradigms

Verbal fluency tests, where only a specific category of item (e.g., animals, words with a specific letter, or verbs) is required to be produced in a limited time (usually within 1 min), produce a funnel effect due to the cognitive constraint of a specific item or category. Two production phases have been described for fluency paradigms^7^: selection (the initial abundant production) and retrieval (slower paced production). Selection occurs approximately from 1 to 15 s, when words are highly available and abundantly produced. Retrieval begins approximately from second 20, when words are less available, and a significant retrieval effort is required to produce only a few words.

### This study

To date, most studies have not performed a sequential-semantic analysis of participant performance in fluency testing. The global score (the total number of words produced) is the most frequent measurement used in fluency testing^8^. However, this scoring criterion does not reflect the production sequence, both in general and in each of the two phases (selection and retrieval), where two different neuropsychological processes are involved. What types of verbs are initially produced? What is the overall production sequence for each subtype of verb? To answer these questions, we performed a sequential analysis on fluency testing based on paper and pencil testing. This study aimed to explore differences in performance between the PD group (PDG) and the comparison group (CG) across three criteria:

- The overall frequency and relative percentage of all MAVs,
- The frequency and relative percentage of each of the three types of MAVs, and
- The sequential production of each of the three types of MAVs.

## METHODS

### Participants

A group of 20 nondemented patients diagnosed with PD (55% males), with a mean age of 66.59 years, and a CG of 20 normal elderly individuals (45% males) matched by age and years of education, with a mean age of 67.74 years, participated in the study. The average number of years of progression after diagnosis was 10.63 (standard deviation=4.71) in PD patients.

The inclusion criteria included a clinical diagnosis of PD. Individuals had to be between 50 and 70 years old and achieve at least 23 score on the Mini-Mental State Examination (MMSE), according to normative data adjusted for low education^9^. Patients should have mild or no symptoms of depression (Montgomery-Asberg Depression Rating Scale^10^, cutoff score=20). All patients were treated with levodopa. Not all Unified Parkinson's Disease Rating Scale data were available for all patients.

The exclusion criteria were diagnosis with an atypical PD syndrome (i.e., Lewy body dementia), symptoms of severe depression, or cognitive impairment.

Patients were diagnosed with PD according to UK Parkinson's Disease Society Brain Bank clinical diagnostic criteria^11^. All patients were at the beginning stage of a protocol to be considered the candidates for surgery to provide deep brain stimulation. The criteria for this protocol included the presence of typical PD accompanied by untreatable motor fluctuations and dyskinesia, and at least 30% motor improvement in the levodopa test. All participants in this study gave their written consent to participate. All data were de-identified using an alpha-numeric code. This research was approved by the ethical committee of the hospital where it was conducted.

### Instruments

#### Mini-Mental State Examination^12^

Mini-Mental State Examination (MMSE) is a brief cognitive assessment that evaluates temporal orientation, spatial orientation, memory, attention, calculation, and language.

#### The Hoehn and Yahr scale^13^

The Hoehn and Yahr (H&Y) Scale is clinical rating scale that defines broad categories of motor function in PD (modified version).

Montgomery-Asberg Depression Rating Scale^8^

This scale evaluates the core symptoms of depression.

#### The Geriatric Depression Scale^14^

This depression scale was used for the CG.

*Verb fluency task*. In this task, participants were asked to follow the instructions: “Tell me as many verbs as you can in 1 min, or words that describe what people do.” No specific instructions were given to generate WBAVs, SBAVs, or InstVs. Responses were recorded by the examiner.

### Procedure

All patients were registered for regular clinical services at the National Institute of Neurology and Neurosurgery (Mexico), clinically diagnosed with PD, and individually evaluated with the cognitive tests in the “on” medication stage; depending on each patient, one or two sessions were needed to complete the evaluation. All results, including the specific sequence of verbs produced by each participant or patient, were stored in databases.

### Fluency analysis

The fluency test results were analyzed by a semantic-sequential approach that focused on the three different types of MAVs: WBAVs, SBAVs, and InstVs. A double-check classification procedure was performed, where two of the authors separately classified the verbs and then compared their classifications. Inter-rater agreement was 98.25. We analyzed the one-by-one sequence of production of each verb, and the motor content of each verb was determined according to San Miguel Abella and González-Nosti^15^.

### Statistical analysis

Descriptive and correlational (nonparametric Spearman's correlation) analyses were performed, focusing on the correlation of clinical measures and cognitive scores. A repeated-measures analysis of variance (ANOVA) was performed to detect differences in the production sequence.

## RESULTS

The participant's demographics and overall results are presented in Table 1. Although the PDG produced a slightly lower number of all verbs on average (lower overall fluency), the difference was not significant from the CG (t-test analysis). Significant differences were found in the overall production of MAVs (the sum of all three motor types), and the PDG presented a lower performance. The InstVs production was the most affected in the PDG (lower than the CG); in contrast, the overall production of WBAVs and SBAVs was not significantly different between the groups.

**Table 1:** Demographic data, mean differences, and ANOVA (repeated measures).

		Control (n=20)	PD (n=20)	Mean difference
Age		67.74 (5.79)	66.59 (4.13)	0.762*
School years		8.23 (2.26)	7.74 (2.90)	0.547*
MMSE		27.14 (2.53)	27.67 (2.42)	0.506*
Sex		14/6	13/8	
Depression†		8.72 (4.96)	4.50 (3.97)	
Years after dx			10.63 (4.71)	
Hoehn and Yahr			2.31 (0.34)	
Fluency	Total verbs	14.59 (4.96)	12.53 (4.42)	0.156*
	All motor	8.68 (3.69)	5.48 (3.37)	0.012*
	WBA	2.81 (1.79)	1.95 (1.59)	0.091‡
	Actions	3.18 (1.81)	2.38 (1.65)	0.223‡
	Instrum	2.68 (2.23)	1.33 (1.60)	0.050‡
ANOVA repeated measures	All motor verbs	Linear model	Quadratic model	
		F=12.007	F=4.839	
		p=0.002	p=0.040	
		ETA=0.364	ETA=0.195	
Covariable effect	School years	F=22.204	F=12.392	
		p=0.000	p=0.002	
		ETA=0.539	ETA=0.395	
	Depression†	F=6.737	F=4.40	
		p=0.018	p=0.049	
		ETA=0.262	ETA=0.188	
Years after dx			Not significant	
Age		Not significant	Not significant	
MMSE score		Not significant	Not significant	

* Mean differences by Student's t-test;

† Depression scores are obtained from different scales;

‡ Median differences by Kruskal-Wallis test, all groups presented similar distributions.

PD: Parkinson's disease group; WBA: whole-body action; Actions: specific actions; Instrum: instrumental actions; F: measurement variability; ETA: effect size; MMSE: Mini-Mental State Examination.

The within-group analysis of the relative percentage of production indicated that the most produced verbs in each group were WBAVs, followed by SBAVs and InstVs. However, the relative percentage of InstVs was different between the groups, with significantly fewer InstVs produced in the PDG. No significant differences were found in the average motor content for each subtype of MAV produced (Table 2). Other types of verbs were produced by participants, but not included in further analysis; these included psychological, emotional, and abstract.

**Table 2:** The most frequent verbs produced (initial production order); the rest of the verbs (up to teen) are presented by the sequence of appearance in the overall production.

	Whole-body actions						Specific actions						Instrumental actions					
	CG	f	MC	PDG	f	MC	CG	f	MC	PDG	f	MC	CG	f	MC	PDG	f	MC
1	Run	13	6.15	Walk	7	4.86	Eat	20	3.55	Eat	11	3.55	Bath	5	3.93	Bath	3	3.93
2	Hop	13	6.0	Run	7	6.15	Sing	8	2.59	Talk	4	3.30	Wash	4	4.23	Shake	3	4.50
3	Jump	9	5.65	Hop	4	6.0	Read	7	2.30	Sing	4	2.59	Write	5	3.15	Sweep	2	4.41
4	Walk	8	4.86	Dance	4	6.35	Talk	6	3.30	Drink	2	3.0	Sweep	3	4.50	Mop	1	4.63
5	Dance	4	6.35	Swim	2	6.41	Greet	3	2.70	See	2	2.22	Brush	3	3.44	Ax	1	5.37
6	Swim	4	6.41	Walk	1	5.65	Drink	3	3.0	Breath	2	2.88	Iron	2	4.38	Cut	1	2.43
7	Climb	1	6.11	Exercise	1	5.19	Knead	2	3.83	Read	1	2.30	Cook	2	4.38	Dress	1	4.46
8	Box	1	6.19	Jump	1	5.65	Shout	2	2.52	Kick	1	5.27	Dress	1	4.46	Drive	1	4.11
9	Walk	1	5.19	Slide	1	4.0	Swallow	1	3.33	Cough	1	3.65	Smoke	1	2.58	Mount	1	4.41
10										Step	1	3.70	Shake	1	4.92	Knit	1	4.31
Fl		54			33			57			36			38			21	
LE	9			9			13			17			15			16		
MCAV			5.87			5.58			3.01			3.24			3.99			4.25

CG: control group; f: frequency; MC: motor content, the degree of the motor component of the verb, the amount of mobility –displacement/movement of the different part of the body, that each actions requires, on a scale of 1 (lower) to 7 (higher); PDG: Parkinson's disease group; Fl: fluency, total verbs produced in the category; LE: lexical span (number of different action names in the category); MCAV: motor content average verb.

### Correlation analyses

Overall fluency correlated with years of education in both groups (CG: r=0.772, p<0.001; PDG: r=0.653, p=0.002), but overall fluency only correlated with the MMSE score in the CG (r=0.429, p=0.046). Overall fluency correlated with all MAVs (CG: r=0.772, p=0.000; PDG: r=0.653, p=0.002) and with SBAVs (CG: r=0.771, p=0.000; PDG: r=0.646, p=0.004) in both groups, but only correlated with WBAVs (r=0.456, p=0.033) in the CG. None of the clinical measures presented statistically significant correlations with fluency test performance. In the PDG, only the years of evolution (after PD diagnosis) correlated with the H&Y scale (r=0.589, p=0.008), and in the CG, only MMSE scores correlated with the depression scores (r=-0.463, p=0.030).

### Sequential analysis

**Figure 1:**
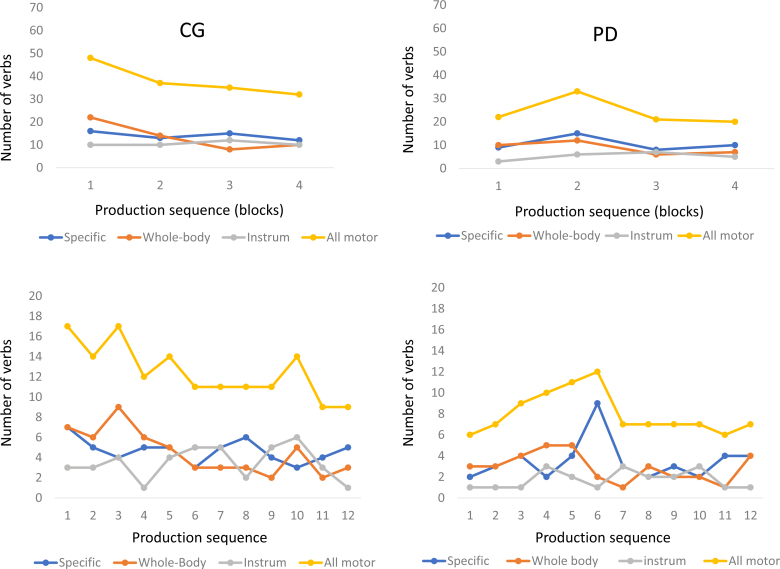
Sequential performance. CG: control group; PDG: Parkinson's disease group.

Based on the PDG performance (an average production of 12 verbs), we decided to analyze three-verb blocks, focusing on the first four blocks produced (Figure 1). In the CG, the initial production of WBAVs was higher and had the highest fluency value for all four blocks. These effects were not found in the PDG.

The groups had different patterns of MAVs production. The CG had a high initial MAVs production with a slow linear-type decay, due to the decline in WBAVs production. In contrast, the PDG had a pyramid-like MAVs production: a slow ascending production with a significantly more rapid decay, due to the combined production of SBAVs and WBAVs. The results of the repeated-measures ANOVA on all motor verbs confirm the linear model for CG production and a quadratic model for PDG production (Table 1). The within-subject effects for the CG and the PDG were all significant.

## DISCUSSION

This study found significant decrements in the production of all types of MAVs in PD patients, which is consistent with the literature^1,2^. The new findings presented here highlight the instrumental category as the least produced in PD patients and at disproportionately lower rates than the CG.

In general, these results suggest that the brain networks that support the production of InstVs may be more compromised in PD. InstVs are not only intransitively complex (agent, object, and recipient of the action) but also require context specification. For example, the verb “to cut” can be used in many different contexts: different cutting tools exist and even the same tools may require different semantic and pragmatic decisions in different real-life contexts^6^. Aging studies on healthy participants have found a significant decrement in usage (mechanical/pragmatic) and semantic/cognitive instrumental knowledge^16^.

Among all MAVs, the InstVs category requires the highest within-network coupling and the most complex cognitive processing decisions^6,16^. Our results suggest that these types of verbs may be the most sensitive to PD motor-related cognitive effects.

The findings of the semantic-sequential analysis indicated a high initial production of WBAVs in the CG but a significantly different and diminished WBAVs production in the PDG. This is due to a different pattern of sequential production between groups (linear vs. quadratic). To the best of our knowledge, these findings have not previously been reported in the literature. The initial high production of WBAVs in the CG may be explained by three main factors: the great majority of these verbs are intransitive (grammatically simple)^17^, they describe frequent everyday actions, and they present high imageability, which is a priming factor in verb processing^18^.

No correlations between clinical or cognitive scores and fluency performance on the PDG were found. Moreover, expected clinical correlations were present (i.e., years of evolution and MMSE scores). Covariable effects (repeated-measures ANOVA) indicate that in both groups, only the education years and the depression scores influenced the sequential performance. However, a higher number of participants is needed to perform more precise covariable statistics.

The semantic-sequential analysis of MAVs presents several new findings:

- The initial production of WBAVs was significantly reduced in the PDG.
- The trajectory of production was different between the groups (quadratic vs. linear).
- Although the lexical span and the motor content were similar in both groups, MAV fluency was altered (diminished) in the PDG, mainly in the dimension of instrumental actions.

Our proposal for the semantic-sequential analysis of motor verbs contributes to more specific data already reported in the literature and deserves further investigation, which may probe the possible advantages as a standard evaluation of fluency performance in motor-related disease.

The main limitation of our study was the small sample size that makes the findings difficult to generalize. Further research is necessary to achieve a wider normative characterization of the aging process on MAV fluency.
